# Erk5 is a mediator to TGFβ1-induced loss of phenotype and function in human podocytes

**DOI:** 10.3389/fphar.2014.00071

**Published:** 2014-04-21

**Authors:** Irbaz I. Badshah, Deborah L. Baines, Mark E. Dockrell

**Affiliations:** ^1^South West Thames Institute for Renal ResearchSurrey, UK; ^2^St. George’s, University of LondonLondon, UK

**Keywords:** TGF-beta, Erk5, podocytes, diabetic nephropathies, barrier function, apoptosis, migration

## Abstract

**Background:** Podocytes are highly specialized cells integral to the normal functioning kidney, however, in diabetic nephropathy injury occurs leading to a compromised phenotype and podocyte dysfunction which critically produces podocyte loss with subsequent renal impairment. TGFβ1 holds a major role in the development of diabetic nephropathy. Erk5 is an atypical mitogen-activated protein (MAP) kinase involved in pathways modulating cell survival, proliferation, differentiation, and motility. Accordingly, the role of Erk5 in mediating TGFβ1-induced podocyte damage was investigated.

**Methods:** Conditionally immortalized human podocytes were stimulated with TGFβ1 (2.5 ng/ml); inhibition of Erk5 activation was conducted with the chemical inhibitor BIX02188 (10 μM) directed to the upstream Mek5; inhibition of Alk5 was performed with SB431542 (10 μM); Ras signaling was inhibited with farnesylthiosalicylic acid (10 μM). Intracellular signaling proteins were investigated by western blotting; phenotype was explored by immunofluorescence; proliferation was assessed with a MTS assay; motility was examined with a scratch assay; barrier function was studied using electric cell-substrate impedance sensing; apoptosis was studied with annexin V-FITC flow cytometry.

**Results:** Podocytes expressed Erk5 which was phosphorylated by TGFβ1 via Mek5, whilst not involving Ras. TGFβ1 altered podocyte phenotype by decreasing P-cadherin staining and increasing α-SMA, as well as reducing podocyte barrier function; both were prevented by inhibiting Erk5 phosphorylation with BIX02188. TGFβ1-induced podocyte proliferation was prevented by BIX02188, whereas the induced apoptosis was not. Podocyte motility was reduced by BIX02188 alone and further diminished with TGFβ1 co-incubation.

**Conclusion:** These results describe for the first time the expression of Erk5 in podocytes and identify it as a potential target for the treatment of diabetic renal disease.

## INTRODUCTION

The rising tide of diabetic nephropathy with all its well documented health and economic consequences are the subject of a great deal of interest to scientists, clinicians, service providers, health service funders, and most importantly patients. There is strong epidemiological evidence asserting that poor glycaemic control is an important risk factor in the development of diabetic nephropathy, yet this clearly is a difficult target to achieve on a large scale (Fioretto et al., 2006). The additional fact that a subset of patients with poor glycaemic control do not develop nephropathy testifies to a more complex nature of the disease. In the absence of targeted education and support to markedly improve glycaemic control in patients with diabetes, targeting downstream mediators of the disease becomes an attractive option.

The powerful pro-fibrogenic growth factor TGFβ1 is involved in a variety of processes that cover apoptosis, migration, adhesion, differentiation, and anti-proliferative responses, but crucially it also plays a pivotal role in the development of diabetic nephropathy where it has been shown to accumulate in injured kidneys (Khalil, 1999; Reeves and Andreoli, 2000; Goldfarb and Ziyadeh, 2001; Siegel and Massagué, 2003; Chuang and He, 2009). The aberrant secretion and activation of TGFβ1 results in excess extracellular matrix (ECM) accumulation through activating the transcription of ECM genes (type I collagen, type IV collagen, fibronectin, and laminin) thereby leading to glomerulosclerosis, and further to the direct stimulation of ECM proteins TGFβ1 has a key role in causing the secretion of additional pro-fibrotic growth factors such as connective tissue growth factor (CCN2/CTGF) which compounds the matter (Ziyadeh et al., 1994; Phanish et al., 2005; Niculescu-Duvaz et al., 2007). The potent pleiotropic TGFβ1 cytokine can alter cell phenotype with the consequence of impairing organ function through mechanisms regulating intercellular communication by altering the expression of the cadherin class of junctional proteins (Maeda et al., 2005), as well as by changing cell morphology via directly regulating the actin cytoskeleton (Vardouli et al., 2008). Critical components of the diabetic milieu, glucose, and advanced glycation end-products (AGEs), induce TGFβ1 expression in human diabetic nephropathy (Iwano et al., 1996) as well as in mesangial cells (Ziyadeh et al., 1998; Kim et al., 2001) and proximal tubule epithelial cells in culture (Rocco et al., 1992). Furthermore, podocytes increase TGFβ1 expression in diabetic glomerulosclerosis and in animal models of diabetic renal disease (Wahab et al., 2005).

The podocyte is a unique cell type which can be seen as a specialized renal pericyte. Resting on the glomerular basement membrane their interdigitating foot processes encapsulate the capillaries of the glomerulus. Formed between these extensions exists a specialized intercellular junction termed the slit diaphragm that is composed of a multitude of proteins including nephrin, P-cadherin, and podocalyxin which imbues the podocyte with its size- and charge-selective nature and thereby functions as a critical regulator of glomerular filtration (Pavenstädt et al., 2003). In recent years considerable attention has focused on the role of podocytes in diabetic nephropathy and mounting evidence suggests that they play a key role in the disease (Li et al., 2007; Herbach et al., 2009). The widening of foot processes and nephrin loss in response to diabetic stimuli results in foot process effacement and podocyte detachment (Binder et al., 1999; Li et al., 2007). Moreover, the podocytopaenia observed in diabetic nephropathy is due to podocyte apoptosis in which TGFβ1 is crucially culpable where it induces apoptosis of cultured murine podocytes (Schiffer et al., 2001). Although podocyte loss in urine has been detected in patients with diabetes, there is some controversy whether absolute podocyte number is important rather than podocyte density (Dalla Vestra et al., 2003). Normally a terminally differentiated cell type, mature podocytes do not proliferate and are unable to reconstitute the loss that is observed in disease; however, maladaptive podocyte proliferation has been detected in different renal pathologies such as cellular focal segmental glomerulosclerosis and has been reported to be stimulated by TGFβ1 *in vitro* (Li et al., 2007).

The multiple and varied effects of TGFβ1 are, in part, related to the variety of intracellular signaling cascades that are stimulated following the binding of TGFβ1 to its heterotetrameric receptor complex which is formed from the dimerisation of type I and type II receptors, namely Alk5 and Alk1 (Huang and Chen, 2012). The canonical signaling that follows is mediated by the Smad family of signaling molecules (Smad2/3 and Smad4), however, this pathway has proved difficult to target with small molecule inhibitors and hence attention has moved to non-canonical signaling such as the mitogen-activated protein (MAP) kinases. The atypical MAP kinase Erk5 lies central to a variety of fundamental cellular processes including cell survival, proliferation, differentiation, and motility which overlap with the cellular processes that become defective in diabetic nephropathy (Drew et al., 2012). Erk5 activity was found to be modified in the glomeruli of an animal model of type 2 diabetes mellitus indicating its dysregulation in disease (Suzaki et al., 2004). In 2008 our group reported the activation of Erk5 by TGFβ1 in human renal tubule epithelial cells (Browne et al., 2008), and in the same year Dorado et al. (2008) reported that TGFβ1-induced collagen I production in mesangial cells was regulated by Erk5. To date there exists no published data on the expression of Erk5 in podocytes along with its possible involvement in TGFβ1-mediated signaling in these cells and the potential role it may play in diabetic nephropathy.

## MATERIALS AND METHODS

### PODOCYTE CULTURE

Conditionally immortalized human podocytes retrovirally transfected with the temperature-sensitive SV40 large T antigen gene (Saleem et al., 2002) were routinely cultured as a monolayer at the permissive temperature of 33°C in a humidified atmosphere of 5% CO_2_ and 95% air, with RPMI Medium 1640 (Life Technologies) supplemented with heat-inactivated FBS (10% v/v; Life Technologies), D-glucose (5 mM; VWR), insulin (5 μg/ml)-transferrin (5 μg/ml)-sodium selenite (5 ng/ml) media supplement (ITS; Sigma–Aldrich), and L-glutamine (2 mM)-penicillin (100 U/ml)-streptomycin (100 μg/ml) solution (PSG; Sigma–Aldrich) with media changes occurring thrice weekly. Once confluent, cells were sub-cultured by aspirating media and incubating at 33°C with 0.5% trypsin-EDTA solution [trypsin (5 g/l), Na2-EDTA (2 g/l), NaCl (8.5 g/l); Life Technologies] for 4 min. The trypsin was neutralized with normal culture media and cells were centrifuged at 350 × *g* for 6 min at room temperature. The cell pellet was re-suspended in culture media; viable cells were counted using 0.4% trypan blue solution (Sigma–Aldrich) and were seeded at a density of 10,000 cells/cm^2^ into cell culture-ware (BD Falcon). Podocytes were terminally differentiated by incubating at the non-permissive temperature of 37°C for 14 days in a humidified atmosphere of 5% CO_2_ and 95% air with RPMI 1640 culture media (supplemented as above). All experiments were conducted in serum-free conditions on terminally differentiated podocytes between passages 3 and 25 that had been subjected to serum starvation overnight.

### STIMULI AND INHIBITORS

Cells were treated at 37°C with the growth factor TGFβ1 (2.5 ng/ml in serum-free media containing BSA (0.1% w/v); Sigma-Aldrich). Inhibitors were applied at 37°C in serum-free media. To diminish Erk5 activation the upstream activator Mek5 was chemically inhibited by BIX02188 (10 μM) with an additional 60 min pre-incubation. TGFβ1-mediated signaling was stopped with SB431542 (10 μM; Sigma–Aldrich), targeting the type I TGFβ receptor Alk5, with a further 30 min pre-incubation. Transmembrane receptor-induced Ras function was prevented with an additional 30 min pre-incubation using farnesylthiosalicylic acid (FTS; 10 μM; Cambridge Bioscience). Controls (vehicles) were treated with serum-free media containing dimethyl sulfoxide (DMSO; 0.1% v/v; Sigma–Aldrich) and BSA (0.1% w/v).

### CELL PROLIFERATION

The extent of cell proliferation was assessed using the colorimetric CellTiter 96 AQ_ ueous_ Non-Radioactive Cell Proliferation Assay (Promega). Cells were cultured in a 96-well plate (BD Falcon) and incubated with the appropriate treatments in a total of 100 μl of serum-free media. At the 48 h experiment end-point 1 ml of PMS [PMS (0.92 mg/ml) in Dulbecco’s PBS (DPBS); KCl (0.2 g/l), NaCl (8.0 g/l), KH_2_PO_4_ (0.2 g/l), Na_2_HPO_4_ (1.15 g/l), MgCl_2_ (100 mg/l)·6H_2_O, CaCl_2_ (133 mg/l)·2H_2_O; pH 7.35] Solution was added to 20 ml MTS Solution and 20 μl of this mixture was then combined with each well containing cells, thereby producing final concentrations of MTS (333 μg/ml) and PMS (25 μM). The cells were then incubated for 2 h at 37°C in a humidified atmosphere of 5% CO_2_ and 95% air. Thereafter, the absorbance at 490 nm was measured with the Sunrise microplate absorbance reader (Tecan) using the accompanying XFluor4 software (v4.51; Tecan); the amount of the red formazan product is directly proportional to the number of living cells. Results were blanked against control wells without cells containing 100 μl serum-free media and 20 μl combined MTS/PMS solution.

### CELL MOTILITY

The assessment of cell motility was conducted using a scratch assay. Initially prior to differentiation, cells were allowed to proliferate to achieve a confluency greater than usual (>70%). After overnight serum starvation culture media was removed and cells were subjected to 60 min pre-incubation with BIX02188 (10 μM) or vehicle and media removed once again. A sterile 200 μl pipette tip was utilized with a smooth sweep across the monolayer. Cells were washed twice in media to remove debris, treatments applied, and 0 h baseline images taken with light microscopy (Nikon). Experiments were concluded by taking light microscopy images and were then examined in ImageJ (v1.46; National Institute of Health). Color images were converted to eight-bit gray scale and the denuded area calculated with the “Polygon Selection Tool”; results were normalized to area values at 0 h with a reduction in area indicating increased motility due to cells migrating into the clear zone.

### BARRIER FUNCTION

Electric cell-substrate impedance sensing (ECIS) was used to investigate barrier function. Monolayers of cells were grown in a 96 well ECIS array: cell culture-ware with a base comprised of gold film electrodes (20 electrodes/well) delineated with an insulating film (Applied Biophysics). After treatment application the ECIS array was seated in the ECIS Z instrument (Applied Biophysics) housed at 37°C in a CO_2_ incubator with a humidified atmosphere of 5% CO_2_ and 95% air. An alternating current (*I*) of 4000 Hz was applied across the electrodes and the ensuing electric potential difference (*V*) created was recorded once per minute, from which the impedance (*Z*) was calculated according to Ohm’s law: *Z* = *V* / *I*. Cells covering the electrode surface perform as insulators and an increase in the distance between adjacent cells caused by dysregulation of intercellular junctions decreased the impedance witnessed which was used as a measure of barrier function; results were normalized to baseline values recorded at 0 h.

### APOPTOSIS

Apoptosis was assessed by flow cytometry with the TACS Annexin V-FITC Kit (Trevigen) and necrotic cells were detected using 7-Amino-Actinomycin D (7-AAD; BD Biosciences). After 48 h of treatment, culture media was aspirated to collect dead cells and dispensed into sterile 1.5 ml microcentrifuge tubes (Appleton Woods). Dying as well as viable cells still adherent were incubated at 37°C with 0.5% trypsin-EDTA solution for 4 min, after which the trypsinised cells were combined with the corresponding culture media supernatants and FBS (1% v/v) was added. Cells were then centrifuged at 400 × *g* for 6 min at 4°C. To remove EDTA, which would otherwise cause chelation of the calcium necessary for binding of annexin V and phosphatidylserine, the cells were re-suspended in 500 μl ice-cold PBS + FBS (1% v/v) and once more centrifuged at 400 × *g* for 6 min at 4°C. After discarding the supernatant cells were re-suspended in 100 μl Annexin V Incubation Reagent [Binding Buffer (1×), 7-AAD (1 μg), Annexin V-FITC (1:10), in deionised H_2_O] for 30 min. Thereafter, 400 μl Binding Buffer (1x) was added and 10,000 cells/sample were counted with a FACSCalibur flow cytometer (BD Biosciences) using the CellQuest Pro software (v5.2.1; BD Biosciences).

### WESTERN BLOTTING

Protein lysates were prepared on ice by aspirating the medium and adherent cells were then rinsed in ice-cold PBS, removed with a cell scraper in 70 μl lysis buffer [Tris-HCl (20 mM), pH 7.5, NaCl (150 mM), EDTA (1 mM), Triton X-100 (1% v/v; Sigma–Aldrich), sodium deoxycholate (0.5% w/v), SDS (0.1% w/v), protease inhibitor cocktail (1×; Roche), phosphatase inhibitor cocktail (2×; Roche)] and lysed with a vortex mixer for 10 s. Lysates were kept on ice for more than 15 min before centrifugation at 10,000 × *g* for 10 min at 4°C after which the supernatant containing protein was aspirated and stored at –80°C. Protein concentrations were determined with a colorimetric bicinchoninic acid (BCA) protein assay kit (Pierce) and absorbance was measured at 562 nm after incubating at 37°C for 30 min with the Sunrise microplate absorbance reader using the XFluor4 software.

Protein samples were prepared for western blotting under denaturing and reducing conditions by heating at 70°C for 10 min in a solution containing NuPAGE LDS Sample Buffer (1×; Life Technologies) and NuPAGE Sample Reducing Agent (1×; Life Technologies). Equal amounts of total protein were resolved by size with electrophoresis on NuPAGE Novex 4–12% Bis-Tris gels (Life Technologies) with NuPAGE MOPS SDS Running Buffer (Life Technologies) using the XCell SureLock Mini-Cell tank (Life Technologies) for 55 min at 200 V constant. Proteins were then transferred onto Immobilon-P PVDF transfer membranes (Merck Millipore) for 4 h at 30 V constant in NuPAGE Transfer Buffer (Life Technologies) using the XCell II Blot Module (Life Technologies).

Post-transfer, membranes were washed for 10 min in tris-buffered saline-Tween-20 [TBS-T; tris base (20 mM), NaCl (137 mM), pH 7.6, Tween-20 (0.1% v/v; VWR)], blocked for 1 h in dried skimmed milk (5% w/v) in TBS-T followed by 3 × 5 min washes in TBS-T. Membranes were then incubated overnight at 4°C with the following primary antibodies: Erk5 (1:1000; New England Biolabs), Phospho-Erk5 (Thr218/Tyr220) (1:1000; New England Biolabs), Phospho-p44/42 MAP kinase (Erk1/2) (Thr202/Tyr204) (197G2) (1:1000; New England Biolabs); the loading control α-/β-tubulin (1:1000; New England Biolabs) was incubated at room temperature for 1 h. After a TBS-T wash procedure of 10 min and then 3 × 5 min, membranes were incubated at room temperature on a 3D gyratory rocker for 1 h with the Anti-Rabbit IgG HRP-linked Antibody (1:2000; New England Biolabs), and were then washed in TBS-T for 10 min followed by 3 × 5 min. Membranes were incubated for 5 min with Solution A:Solution B (1:1) of the Amersham ECL Prime western blotting detection reagent kit (GE Healthcare Life Sciences). The membranes were then exposed to Amersham Hyperfilm ECL (GE Healthcare Life Sciences) films and subsequently developed using the MI-5 X-ray film processor (VWR). Western blot films were imaged with the ImageQuant 300 imager and ImageQuant Capture software (v1.0.0.4; GE Healthcare Life Sciences). Quantification was conducted by performing densitometry with the ImageQuant TL software (v1.1.0.4; GE Healthcare Life Sciences) where target protein band intensity was expressed relative to that of the corresponding loading control.

### IMMUNOFLUORESCENCE

At the experimental end-points, media was aspirated and cells were washed with warm PBS. Cells were then fixed and permeabilised with ice-cold methanol for 15 min at –20°C followed by removing the methanol and rehydrating in PBS for 15 min. Cells were incubated in blocking buffer [Triton X-100 (0.2% v/v), serum (5% v/v) derived from the species of the primary antibody in PBS] for 1 h at room temperature before being rinsed in PBS and incubating overnight at 4°C with the following primary antibodies: Human/Mouse Erk5/BMK1 (15 μg/ml; R&D Systems), P-Cadherin (1:100; Insight Biotechnology), Monoclonal Anti-α-Smooth Muscle Actin Clone 1A4 (1:200; Sigma–Aldrich), Synaptopodin (H-140; 1:50; Insight Biotechnology), diluted in primary antibody dilution buffer [Triton X100 (0.2% v/v) in PBS]. The following day cells were subjected to 3 × 5 min washes in PBS prior to incubating in darkness at room temperature for 1 h with the following fluorochrome-conjugated secondary antibodies diluted in secondary antibody dilution buffer [Triton X-100 (0.2% v/v), and DAPI in PBS]: Alexa-Fluor 488 Donkey Anti-Goat IgG (H+L) (1:200; Life Technologies), Alexa-Fluor 555 Goat Anti-Mouse IgG (H+L) (1:200; Life Technologies), Alexa-Fluor 555 Goat Anti-Rabbit IgG (H+L) (1:200; Life Technologies). Cells were washed for 3 × 5 min in PBS and subsequently covered in mounting buffer [90% glycerol (Sigma–Aldrich) in de-ionized H_2_O] before examination with an inverted fluorescent microscope (Nikon) at the appropriate excitation wavelength using the NIS-Elements imaging software (Nikon). Cells were stored at 4°C protected from light.

### STATISTICAL ANALYSIS

Quantitative data are presented as means ± standard error of the mean (SEM). Statistical analysis was conducted using one-way analysis of variance (ANOVA) with Tukey’s multiple comparison *post hoc* test when more than two groups were being analyzed simultaneously and when two groups were being analyzed a paired *t*-test was conducted with the statistical software GraphPad Prism. Differences were considered statistically significant at values of *P* < 0.05 denoted by a single asterisk (*), *P* < 0.01 denoted by a double-asterisk (** and *P* < 0.001 denoted by a triple-asterisk (***).

## RESULTS

### Erk5 EXPRESSION IN HUMAN PODOCYTES

**FIGURE 1:**
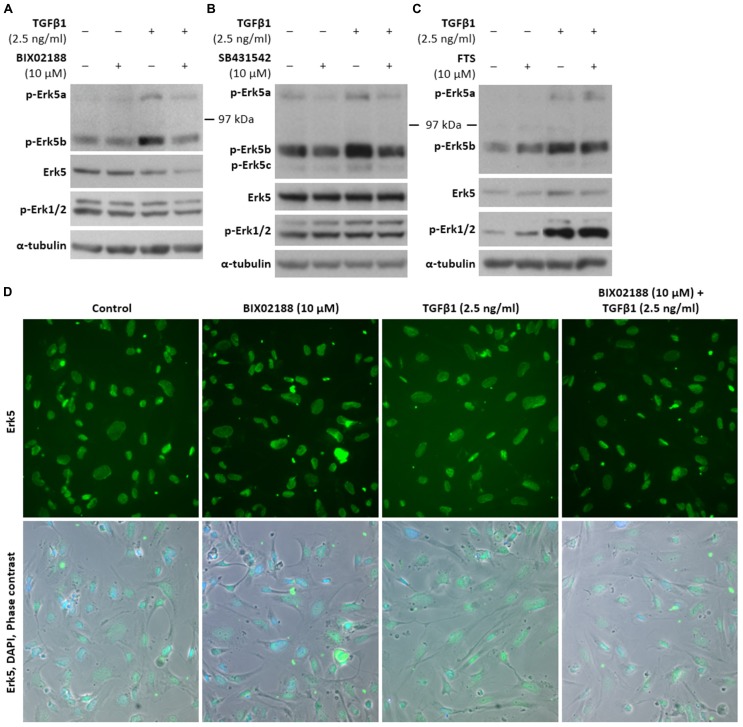
**Erk5 expression, activation, and subcellular localisation in podocytes via TGFβ1. (A)** Representative western blot of p-Erk5, Erk5, and p-Erk1/2 protein expression in conditionally immortalized human podocytes pre-incubated for 60 min with the Mek5 inhibitor BIX02188 (10 μM) followed by co-incubation with TGFβ1 (2.5 ng/ml) for 5 min. Podocytes expressed Erk5 protein and TGFβ1 significantly increased protein levels of p-Erk5b (*P* < 0.05), whilst BIX02188 co-treatment reduced Erk5 activation in response to TGFβ1 (*P* < 0.05); Erk5 was used as a loading control; paired *t*-test, *n* = 3. Levels of p-Erk1/2 did not significantly decrease with BIX02188 co-treatment relative to the TGFβ1 stimulus; α-/β-tubulin was used as a loading control; one-way ANOVA with Tukey’s multiple comparison *post hoc* test, *n* = 3. **(B)** Representative western blot of p-Erk5, Erk5, and p-Erk1/2 protein expression in podocytes pre-incubated for 30 min with the Alk5 inhibitor SB431542 (10 μM) followed by co-incubation with TGFβ1 (2.5 ng/ml) for 5 min. SB431542 decreased p-Erk5 levels both in non-stimulated and TGFβ1-stimulated cells (*P* < 0.05); Erk5 was used as a loading control; paired *t*-test, *n* = 3. **(C)** Representative western blot of p-Erk5, Erk5, and p-Erk1/2 protein expression in podocytes pre-incubated for 30 min with the Ras inhibitor FTS (10 μM) followed by co-incubation with TGFβ1 (2.5 ng/ml) for 5 min. Levels of p-Erk5 did not change significantly following Ras inhibition; Erk5 was used as a loading control; paired *t*-test, *n* = 3. **(D)** Immunofluorescence staining of Erk5 in podocytes pre-incubated for 60 min with BIX02188 (10 μM) followed by co-incubation with TGFβ1 (2.5 ng/ml) for 5 min. Erk5 was mainly, but not exclusively localized to the nucleus and BIX02188 treatment reduced the diffuse staining present in the cytoplasm. TGFβ1 stimulation increased the diffuse cytoplasmic staining and following BIX02188 co-treatment this was reduced which additionally slightly reduced nuclear Erk5 staining.

Erk5 protein expression was investigated due to the absence of any published previous investigation. Indeed, western blotting revealed the expression of Erk5 in conditionally immortalized human podocytes (**Figure 1A**). Following 5 min TGFβ1 (2.5 ng/ml) treatment at 37°C in the absence of serum podocytes exhibited three bands recognized by a phospho (p)-Erk5 antibody (Thr218/Tyr220) at ~120, ~80, and ~75 KDa, which paralleled the three splice variants of Erk5 that exist: Erk5a, Erk5b, and Erk5c; expression was dependent on the upstream Mek5 as 60 min pre-incubation and co-treatment with the Mek5 chemical inhibitor BIX02188 (10 μM) prevented Erk5 phosphorylation (**Figure 1A**). BIX02188 at 10 μM had no effect on p-Erk1/2 in these cells. Immunofluorescence depicted the basal subcellular localization of Erk5 to be predominantly nuclear and BIX02188 reduced the diffuse staining present in the cytoplasm (**Figure 1D**). Stimulation with TGFβ1 for 5 min increased the cytoplasmic staining of Erk5 (**Figure 1D**). BIX02188 5 min co-treatment reduced Erk5 cytoplasmic staining and to lesser extent nuclear staining as evinced by a greater proportion of cells with nuclei that predominantly exhibited DAPI staining over Erk5 staining (**Figure 1D**). TGFβ1-induced Erk5 activation at 5 min was Alk5-dependent as a 30 min pre-incubation and 5 min co-incubation with the TGFβ type I receptor inhibitor SB431542 (10 μM) reduced p-Erk5 levels (**Figure 1B**). However, pre-incubation for 30 min and subsequent co-incubation with FTS (10 μM) had no effect, demonstrating no involvement of the small GTPase Ras (**Figure 1C**).

### PODOCYTE PROLIFERATION

**FIGURE 2:**
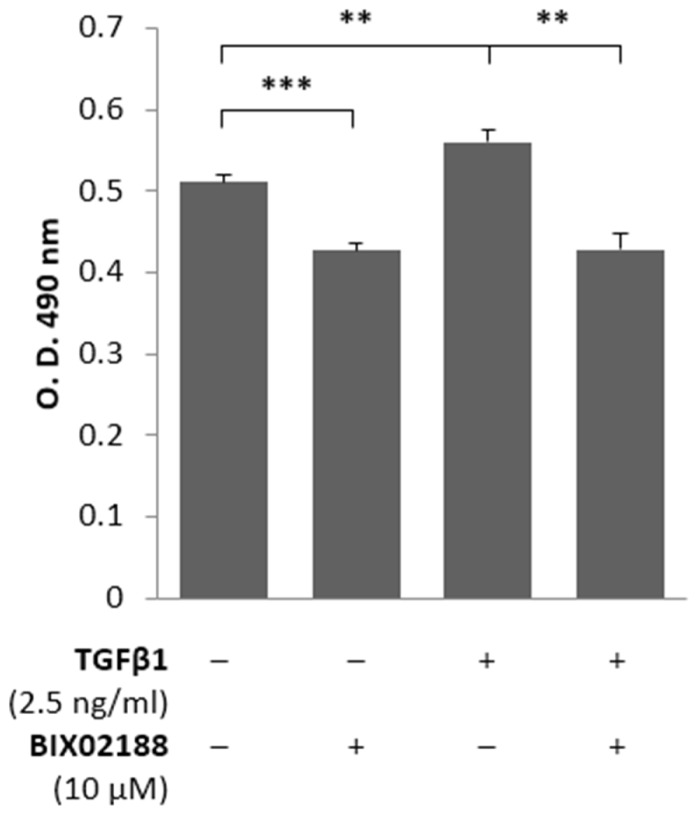
**Regulation of podocyte proliferation via Erk5.** Colorimetric cell proliferation assay investigating proliferation of podocytes pre-incubated for 60 min with the Mek5 inhibitor BIX02188 (10 μM) followed by co-incubation with TGFβ1 (2.5 ng/ml) for 48 h. TGFβ1 induced podocyte proliferation which was prevented with BIX02188 co-treatment. **P < 0.01, ***P < 0.001; paired *t*-test, *n* = 6.

Podocytes are terminally differentiated cells that do not proliferate under normal physiological conditions. To assess the proliferation of podocytes in response to the pro-fibrotic stimulus of TGFβ1, podocytes were pre-incubated in the presence and absence of BIX02188 (10 μM) for 60 min after which cells were co-treated with TGFβ1 (2.5 ng/ml) for 48 h to provide adequate time for proliferation to occur and a colorimetric cell proliferation assay was employed where metabolic activity is directly proportional to cell number. Inhibition of Erk5 activation with BIX02188 incubation reduced podocyte cell number (**Figure 2**). TGFβ1 stimulation increased podocyte cell number which was prevented following BIX02188 co-treatment (**Figure 2**).

### PODOCYTE MOTILITY

**FIGURE 3:**
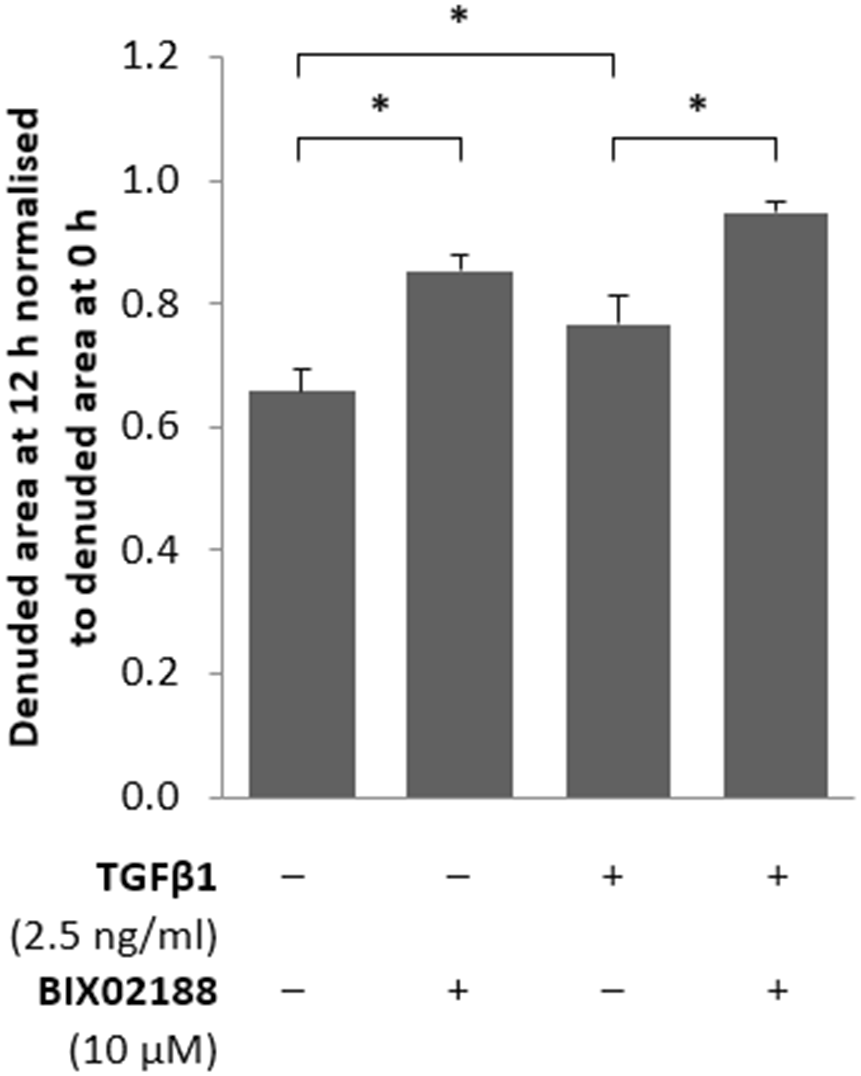
**Regulation of podocyte motility via Erk5.** Podocytes pre-incubated for 60 min with the Mek5 inhibitor BIX02188 (10 μM), after which the monolayer was scratched and subsequently co-treated with TGFβ1 (2.5 ng/ml) for 12 h. Inhibition of Erk5 activity decreased podocyte motility as did TGFβ1 treatment and co-treatment further augmented the reduction in motility observed; data normalized to the denuded area measured at 0 h. *P < 0.05; paired *t*-test, *n* = 3.

An investigation into the involvement of Erk5 in regulating podocyte motility was conducted with a scratch assay where cells were pre-incubated for 60 min with BIX02188 (10 μM) and subsequently cells were removed from a confluent monolayer and the extent of cell migration into the denuded area was calculated following treatment with TGFβ1 (2.5 ng/ml) for 12 h as this provides sufficient time for migration to occur without the recorded area reaching saturation. TGFβ1 stimulation for 12 h reduced podocyte motility, as did Mek5 inhibition, relative to control treated cells that exhibited migration (**Figure 3**).

### PODOCYTE BARRIER FUNCTION

**FIGURE 4:**
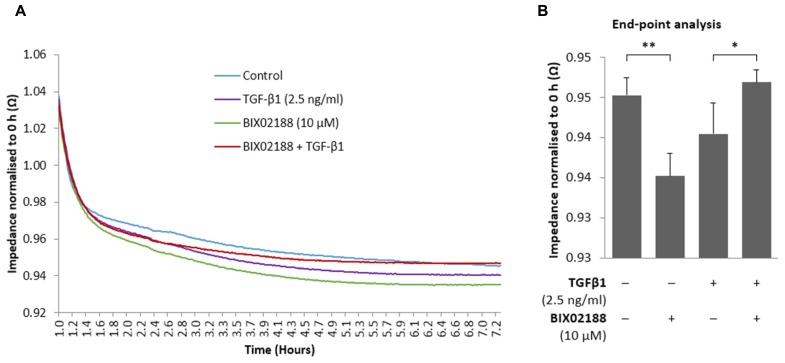
**Regulation of podocyte barrier function via Erk5. (A,B)** Podocytes pre-incubated for 60 min with the Mek5 inhibitor BIX02188 (10 μM) followed by co-incubation with TGFβ1 (2.5 ng/ml) for 6 h and subjected to ECIS where transcellular impedance was measured. TGFβ1 reduced podocyte transcellular impedance. BIX02188 treatment alone decreased barrier function, whereas co-treatment with TGFβ1 significantly increased barrier function thereby restoring it to control levels. **(B)** 6 h end-point representation of ECIS readout; data normalized to baseline values recorded at 0 h. *P < 0.05, **P < 0.01; paired *t*-test, *n* = 6.

To observe the role that Erk5 activity imparts on podocyte barrier function cells were grown on an electrode-coated surface and impedance across the cell layer was measured by ECIS. Podocytes were pre-incubated for 60 min with the Mek5 inhibitor BIX02188 (10 μM) followed by co-incubation with TGFβ1 (2.5 ng/ml) for 6 h to allow morphological changes to initiate whilst avoiding observations that could be attributed to changes in cell number. TGFβ1 reduced transcellular impedance, and Mek5 inhibition decreased barrier function in non-stimulated conditions whilst co-treatment with TGFβ1 restored barrier function to control levels (**Figure 4**).

### PODOCYTE PHENOTYPE

**FIGURE 5:**
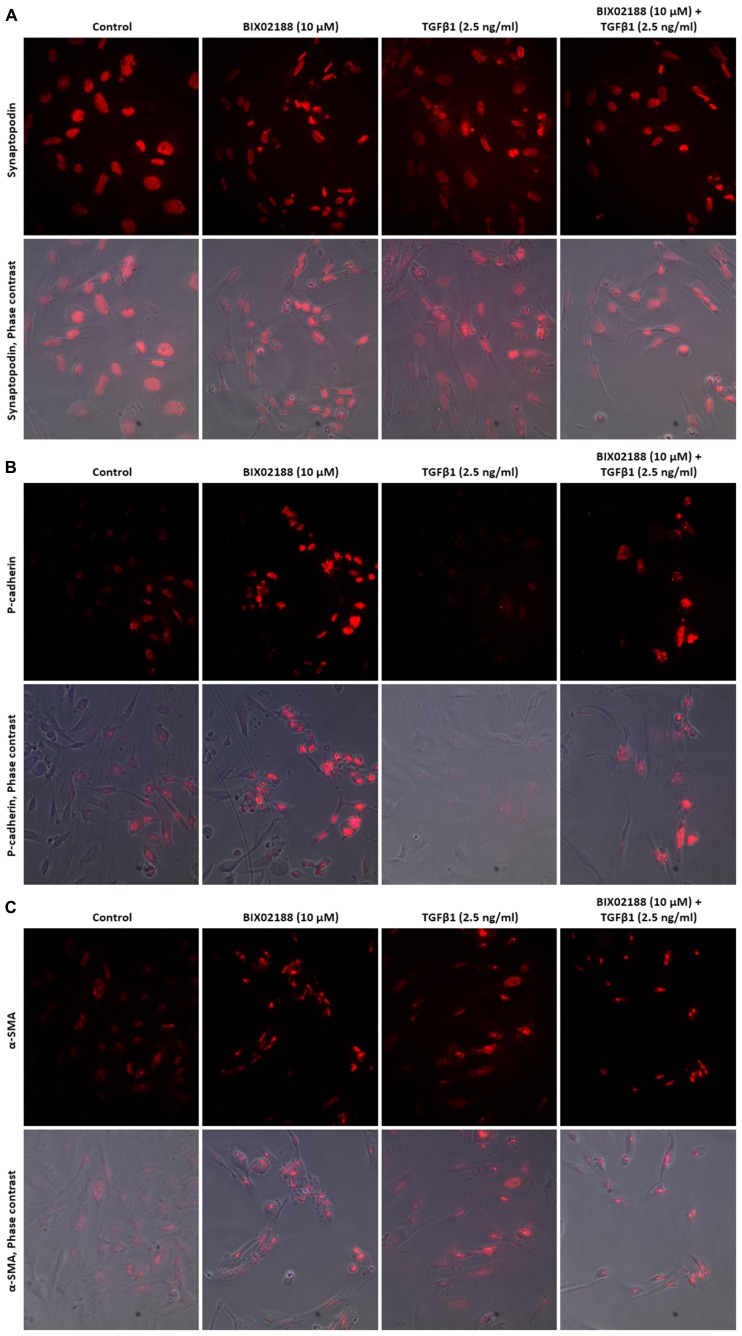
**Regulation of podocyte phenotype with TGFβ1 via Erk5. (A)** Immunofluorescence staining of synaptopodin in podocytes pre-incubated for 60 min with the Mek5 inhibitor BIX02188 (10 μM) followed by co-incubation with TGFβ1 (2.5 ng/ml) for 48 h. Synpatopodin staining was greatest in the nucleus with diffuse cytoplasmic staining. BIX02188 treatment alone did not affect synaptopodin localization, whilst TGFβ1 treatment increased cytoplasmic staining and co-treatment reduced this increase. **(B)** Immunofluorescence staining of P-cadherin in podocytes pre-incubated for 60 min with the Mek5 inhibitor BIX02188 (10 μM) followed by co-incubation with TGFβ1 (2.5 ng/ml) for 48 h. P-cadherin exhibited punctate nuclear staining in the non-stimulated control and TGFβ1 treatment decreased the expression observed. BIX02188 treatment alone increased nuclear staining relative to the control and TGFβ1 co-treatment prevented the TGFβ1-induced reduction. **(C)** Immunofluorescence staining of α-SMA in podocytes pre-incubated for 60 min with the Mek5 inhibitor BIX02188 (10 μM) followed by co-incubation with TGFβ1 (2.5 ng/ml) for 48 h. There was weak punctate nuclear staining of α-SMA and faint diffuse cytoplasmic staining under non-stimulated conditions. BIX02188 treatment alone increased the nuclear staining, whilst also reducing the cytoplasmic staining. TGFβ1 increased the nuclear staining intensity and increased the cytoplasmic staining which was prevented with BIX02188 co-treatment.

The role of Erk5 in TGFβ1-mediated changes in podocyte phenotype was explored with immunofluorescence to study the expression and subcellular localization of the podocyte marker synaptopodin, P-cadherin as an epithelial cell marker and α-smooth muscle actin (α-SMA) as a mesenchymal cell marker. Podocytes were pre-incubated with the Mek5 inhibitor BIX02188 (10 μM) for 60 min followed by subsequent co-incubation with TGFβ1 (2.5 ng/ml) for 48 h. Synaptopodin was concentrated in the nucleus with diffuse staining in the cytoplasm under non-stimulated conditions (**Figure 5A**). BIX02188 treatment alone did not affect synaptopodin subcellular localization, whilst TGFβ1 treatment increased cytoplasmic staining and co-treatment prevented this increase (**Figure 5A**). Podocytes demonstrated punctate nuclear staining of P-cadherin and TGFβ1 treatment decreased the expression observed relative to the non-stimulated control (**Figure 5B**). BIX02188 treatment alone increased nuclear staining relative to the control and TGFβ1 co-treatment prevented the TGFβ1-induced reduction (**Figure 5B**). Under non-stimulated conditions there was weak punctate nuclear staining of α-SMA and faint diffuse staining in the cytoplasm (**Figure 5C**). BIX02188 treatment alone increased the nuclear staining observed, whilst also reducing the cytoplasmic staining (**Figure 5C**). TGFβ1 increased the nuclear staining intensity and greatly increased the cytoplasmic staining which was prevented with BIX02188 co-treatment (**Figure 5C**).

### PODOCYTE APOPTOSIS

**FIGURE 6:**
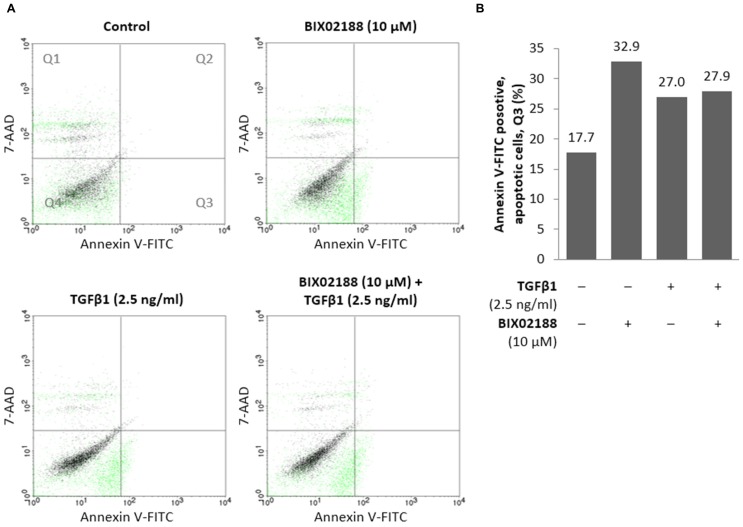
**Regulation of podocyte apoptosis via Erk5. (A,B)** Flow cytometry analysis of podocytes pre-incubated for 60 min with the Mek5 inhibitor BIX02188 (10 μM) followed by co-incubation with TGFβ1 (2.5 ng/ml) for 48 h. Inhibition of Erk5 activation increased podocyte apoptosis which also occurred following TGFβ1 stimulation as well as co-treatment. **(B)** Representation of apoptotic cells located in Q3 seen above.

The loss of podocytes is a key aspect of diabetic nephropathy and a variety of factors have been implicated, most notably TGFβ1 (Dessapt et al., 2009). To explore apoptosis podocytes were pre-incubated with BIX02188 (10 μM) prior to TGFβ1 (2.5 ng/ml) co-treatment for 48 h and subjected to analysis by flow cytometry for the externalization of phosphatidylserine which occurs as part of the apoptotic process. Inhibiting Erk5 activity resulted in an increase in podocyte apoptosis (**Figure 6**). TGFβ1 treatment also produced an increase in podocyte apoptosis as did co-treatment with BIX02188 (**Figure 6**).

## DISCUSSION

Since the first report of Erk5 (Zhou et al., 1995) there has been an ever growing interest in its potential role in physiology and pathology. The conventional Erk5 knock-out mouse demonstrated embryonic lethality, highlighting its importance, and Erk5 was strongly implicated in cardiovascular development and angiogenesis due to a loss of vascular integrity through increased endothelial cell apoptosis (Regan et al., 2002; Hayashi et al., 2004). Subsequently, the critical role for Erk5 in cell survival and proliferation suggested it as a possible target for cancer therapies (Winn et al., 2006). Suzaki et al. (2004) identified a potential role for Erk5 in the pathogenesis of the diabetic kidney describing increased activation of Erk5 in the glomeruli of diabetic rats and using *in vitro* techniques to demonstrate its activation in mesangial cells by high glucose. The significant role that Erk5 might possess in diabetic nephropathy was furthered in later reports by our group and others of TGFβ1-mediated activation of Erk5 in both renal tubule epithelial cells and mesangial cells (Browne et al., 2008; Dorado et al., 2008). However, hitherto the work detailed herein there have been no reports of its expression or activation in podocytes, a cell now considered central in diabetic renal injury.

Here we describe the presence in transformed human podocytes of three splice variants of Erk5 as depicted by the expression of three p-Erk5 protein bands. As in other renal cells we were able to demonstrate the phosphorylation on Thr218/Tyr220, the proposed target for the upstream activator Mek5. We confirmed this by the use of the selective Mek5 inhibitor BIX02188 and also established that the activation was dependent on the TGFβ type I receptor, Alk5. However, it cannot be concluded from our work which isoform or isoforms may be responsible for the specific activities observed. Alternative splice variants from the same gene often have opposing actions and any proposition to manipulate the Mek5/Erk5 axis should be preceded by an investigation involving alternative splicing. Interestingly, TGFβ1-mediated Erk5 activation did not involve Ras as FTS treatment did not alter Erk5 phosphorylation, thus emphasizing the cell type-specific nature of Erk5 signaling given that Ras involvement is inconsistent between a selection of cancer cell lines (Drew et al., 2012).

Podocyte proliferation and apoptosis in response to TGFβ1 has been described previously by Herman-Edelstein et al. (2011), however, their work did not seek to investigate the molecular mechanisms responsible. Here we replicate their observation but further identify that these are by differing cell signaling pathways. TGFβ1-induced proliferation of podocytes was significantly inhibited by blocking Erk5 phosphorylation, however, TGFβ1-induced apoptosis was unaffected. Changes in podocyte phenotype were consistent with the changes in barrier function observed following TGFβ1 treatment, and both were markedly attenuated by Erk5 inhibition; a concomitant reduction of P-cadherin and increased α-SMA with TGFβ1 stimulation is consistent with a transgression of phenotype which was reversed through the inhibition of Erk5 activation. Although controversy surrounds epithelial-to-mesenchymal transition regarding podocytes, Herman-Edelstein et al. (2011) better characterized the process as dedifferentiation. Podocytes are more akin to pericytes rather than being true epithelial cells and the changes induced by TGFβ1 in podocytes are not typical of epithelial-to-mesenchymal transition, specifically the suppression of proliferation and the reduction in tight junction formation was not observed. The unique structure and phenotype of podocytes is what allows them to precisely regulate glomerular barrier function; it is not proposed that the barrier function observed here *in vitro* is exactly the same. Glomerular barrier function *in vivo* is potentially affected by a number of factors, for example the experiments conducted herein did not replicate the pressure of fluid as would be exerted from the blood vessels along with the ensuing signaling that results from shear stress. The loss of the specialized podocyte phenotype that is reported here would be consistent with cytoskeletal changes resulting in the flattening and loss of podocyte foot processes, a phenotype which is associated with proteinuria *in vivo*; this can be witnessed in an elegant time-lapse by Herman-Edelstein et al. (2011). The observed flattening following TGFβ 1 masked the reduction in barrier function detected as determined with ECIS, since the increased cell surface area enhances the impedance conferred by the monolayer, hence the limited reduction with TGFβ 1. With this consideration it would appear from our work that TGFβ1 has the potential to modify the specialized phenotype of the podocyte in a manner likely to alter its function, and consequently still represents a loss of barrier function which can be mitigated to a large extent by inhibiting the activation of Erk5 by Mek5.

One of the early reports of TGFβ-mediated activation of Erk5 indicated that the kinase was required for protein stabilization of the Snail transcription factor (Marchetti et al., 2008), a process known to be involved in the regulation of cell differentiation along with epithelial-to-mesenchymal transition and was recently demonstrated in nephrin loss from podocytes (Gagliardini et al., 2013). The agonist used in the work by Gagliardini et al. (2013) was Angiotensin II, another known activator of Erk5 and a target in diabetic nephropathy. Although our work did not specifically address molecular mediators downstream of Erk5, this could be a potential mechanism in the regulation of podocyte phenotype.

As stated above, diabetic nephropathy is a disease associated with marked podocyte dysfunction, but it is also characterized by extensive renal fibrosis. Consequently, the work by Dorado et al. (2008) describing the role of Erk5 in mediating TGFβ-induced collagen I production in mesangial cells is of considerable interest in the context of our own work; Erk5 may prove to be a key target in controlling the multiple cellular processes that constitute diabetic nephropathy. In pulmonary fibrosis Erk5 also appears to be necessary for the production of ECM by fibroblasts and epithelial cells, however, in this case it is associated with Smad3 acetylation; acetylation of Lys19 on Smad3 enhances the binding of Smad3 to DNA (Kim et al., 2013). The activation of Erk5 by glucose in retinal and vascular endothelial cells causes a significant rise in fibronectin expression, a feature of diabetic retinopathy (Wu et al., 2012). Interestingly, in these experiments the effect of Erk5 may be Smad-independent as expression of constitutively active Erk5 was associated with a decrease in Smad2 phosphorylation. TGFβ-induced fibronectin expression in human renal proximal tubule epithelial cells is also Smad-independent whilst being p38-dependent, thus targeting MAP kinase-mediated control of ECM production would be valuable in disease (Niculescu-Duvaz et al., 2007).

Despite the evidence above, the therapeutic down-regulation of Erk5 remains controversial, perhaps particularly in diabetes largely because of the suspected effects on endothelial cells. It is certainly true that the phenotypes of the embryos from Erk5 knock-out mice demonstrate that Erk5 is vital for the development of the endothelium (Regan et al., 2002). There is also evidence that shear force activates Erk5 in endothelial cells and that this is a protective mechanism inhibiting apoptosis (Shishido et al., 2008). Furthermore, Shishido et al. (2008) asserted that loss of Erk5 transcriptional activity through conjugation of small ubiquitin-like modifier (SUMO) in a mechanism termed SUMOylation, induced at least in part by AGEs resulting from diabetes, is deleterious (Woo et al., 2008). However, the groups do provide evidence to support their assertion that SUMOylation does not alter Erk5 phosphorylation or kinase activity but does inhibit transcription of certain Erk5 targets, such as KLF2. The work suggests that the effect of SUMOylation on Erk5 activation of the KLF2 promoter is independent of Erk5 phosphorylation. However, the current received wisdom is that Erk5 activation of transcription requires phosphorylation; therefore inhibition of Erk5 phosphorylation on Thr218/Tyr220 would also inhibit KLF2 promoter activity and eNOS expression. What is not clear is whether the anti-podocyte effects of TGFβ1 mediated by Erk5 described in this manuscript require transcription, as this was not within the remit of this investigation or whether other mechanisms such as protein stabilization or acetylation may be involved.

## CONCLUSION

These results describe for the first time the expression of Erk5 in podocytes and strongly supports Erk5 as a mediator to TGFβ-induced loss of podocyte phenotype and function and, in the context of other work, potentially demonstrates a pro-fibrotic effect of TGFβ1 being mediated by Erk5 thereby suggesting that Erk5 should be considered a novel target in diabetic renal disease. However, the evidence that loss of certain transcriptional activity of Erk5 may be harmful requires that further investigation is carried out into the precise mechanism in which Erk5 regulates the complex nature of podocyte phenotype and barrier function.

## Conflict of Interest Statement

The authors declare that the research was conducted in the absence of any commercial or financial relationships that could be construed as a potential conflict of interest.
